# Attributional styles are associated with care burden in geriatric depression: older adults and their caregivers in Taiwan

**DOI:** 10.1007/s40520-024-02762-2

**Published:** 2024-05-08

**Authors:** Ching-Yen Chen, Jian‑Hong Chen, Shao‑Chun Ree, Chia‑Hui Chen, Sheng-Hsiang Yu

**Affiliations:** 1 Department of Psychiatry, Chang Gung Hospital, Keelung, Taiwan; 2 Faculty of Medicine, Chang Gung University, Taoyuan, Taiwan; 3 Department of Nursing, Chang Gung Hospital, Keelung, Taiwan; 4 Present Address: Department of Psychology and Counseling, National Taipei University of Education, No. 134, Sec. 2, Heping E. Rd., Da’an Dist, Taipei, Taiwan

**Keywords:** Caregiver, Care burden, Older adults, Geriatric depression, Attributional style

## Abstract

**Background:**

Given the rising prevalence of depression among older adults and the associated increase in caregiving responsibilities, understanding factors influencing caregiver burden is crucial. Previous research has not extensively explored the impact of caregivers’ attributional styles, that is, how individuals interpret the causes of life events, on their care burden.

**Aim:**

This study examined the relationship between caregivers’ attributional styles and their care burden for older patients with depression.

**Methods:**

This cross-sectional study enrolled older adults aged ≥ 65 years diagnosed with depression and their caregivers. Depression was diagnosed according to the DSM-V criteria for Major Depressive Disorder or Persistent Depressive Disorder. Caregivers completed the Chinese Depression Caregiver Burden Scale (CDCBS) to assess care burden, the Hamilton Depression Rating Scale (HAM-D) to evaluate patient symptom severity, the Center for Epidemiological Studies Depression Scale (CES-D) for measuring caregivers’ depression, and the Chinese Depression Patient Caregiver Attribution Style Scale (CDPCAS) to assess attributional styles. Hierarchical regression analysis was used to identify the factors independently associated with the caregiver’s subjectively assessed care burden.

**Results:**

The sample included 146 caregivers of geriatric patients with depression. Most depression patients were women (74.7%) with a mean age of 74.3 years, whereas the mean age of caregivers was 57.7 years. Hierarchical regression analysis identified that caregivers’ gender (*β* = − 0.14, *p* = .044), educational level (*β* = 0.19, *p* = .008), caregivers’ own depression assessed by the Center for Epidemiological Studies Depression Scale (*β* = 0.41, *p* < .001), and attributional styles, particularly manipulation (*β* = 0.29, *p* < .001) and illness/stress attributional style (*β* = 0.23, *p* = .002) as independent factors associated with care burden. Patient symptom severity assessed using the Hamilton Depression Scale was not significantly correlated with care burden after controlling for attributional styles.

**Conclusions:**

Certain attributional styles, particularly the manipulation and illness/stress attributional styles, significantly increased self-reported care burden. These findings highlight the need for educational resources to change the attribution style, along with support systems and accessible mental health services for caregivers to potentially ease the care burden.

**Supplementary Information:**

The online version contains supplementary material available at 10.1007/s40520-024-02762-2.

## Background

Depression is a globally prevalent mental health condition, causing financial burden and reducing the quality of life of individuals and societies. In 2020, there were approximately 3,153 cases of major depression per 100,000 people globally [1]. In Taiwan, the prevalence of depression as a public health concern has increased from 1.61% in 2007 to 1.92% in 2016 [2]. In a nationwide sample from Taiwan, the prevalence rates of minor and major depression among individuals aged 55 years and older were 3.7% and 1.5%, respectively [3]. Depression frequently remains undiagnosed in older adults [4]. Despite their higher healthcare utilization compared to younger adults, older individuals may be reluctant to seek mental health care because of social stigma or limited awareness [5]. Therefore, the actual prevalence of depression among older adults has likely been underestimated.

*Caregiver burden* refers to the stress related to caring for family members who are chronically ill, disabled, or older. This results in diminished caregiving effectiveness and negatively impacts the caregivers’ physical and mental health [6]. The burden of caring for individuals with depression is particularly challenging and often leads to psychological stress and financial strain [7]. The burden of caregiving heightens depressive symptoms among caregivers, which in turn influences their attitudes and behaviors. Consequently, caregivers may develop feelings of rejection and frustration towards patients [8]. Understanding the factors associated with care burden is crucial for optimal resource distribution and for ensuring that adequate healthcare assistance and services are accessible to those in need.

*Attributional style*, a psychological aspect of personality, describes how one interprets the underlying causes of life’s favorable or unfavorable events [9]. The application of attributional style has recently been extended to explore its relationship to mental health [10–12]. Previous studies have highlighted that specific attribution styles such as internal, intentional, responsibility, and controllable behaviors are linked to poorer relationship satisfaction and heightened caregiver burden, particularly among younger caregivers [13]. Notably, character attributions, which involves attributing patients’ behaviors to their personality traits, have been consistently linked to increased burden [14]. This suggests that caregivers employing character attributions may perceive caregiving as unnecessary, leading to greater strain. Furthermore, caregivers may attribute responsibility for patients’ symptoms to the patients themselves due to perceived intentionality in their behaviors [15]. However, the broader implications of diverse attribution styles for caregivers of older adults with depression remain under explored.

Therefore, this study aimed to examine the relationship between caregivers’ attributional styles, other critical factors including caregiver’s demographics, patients’ symptom severity, caregivers’ depression level and the burden of caring for older patients with depression.

## Methods & materials

### Study design and selection criteria

This cross-sectional survey included patients aged 65 years or older diagnosed with depression, along with their caregivers, who were receiving psychiatric services at a single center in southern Taiwan between August 2020 and July 2021. Diagnoses of depression were based on the *Diagnostic and Statistical Manual of Mental Disorders, Fifth Edition* (DSM-V) criteria for Major Depressive Disorder (MDD) or persistent depressive disorder (PDD) [16].

The enrolled patients were provided with detailed information about the study, including its purpose and the procedures involved, before they signed the informed consent form. All survey items were completed on paper, and the relevant research personnel or assistants individually completed the surveys with the patients to ensure they understood the meaning of the survey questions. The survey took approximately 20–30 min to complete the questionnaire. Participants received NT$ 100 as compensation for their time.

### Caregivers

Individuals were identified as caregivers if they fulfilled more than two of the eligibility criteria: (a) being in the company of the patient for more than two hours a day; (b) having meals with the patient more than four times a week; (c) engaging in leisure activities with the patient, such as going to movies or taking a walk together, more than once a week; and (d) helping a patient with daily activities, such as taking medicine, more than four times a week on average over the previous year. Additional inclusion criteria were: (1) 20 years of age or older; (2) having a non-employed relationship with the patient with depression as a relative or friend. (e.g., parent, spouse, child, sibling, friends; and 3) must be aware of the patient’s diagnosis of depression. A total of 146 caregivers met the inclusion criteria and completed a self-administered questionnaire when they accompanied patients with depression to the hospital. The questionnaire measured sociodemographic characteristics, care burden, depressive symptoms, and the evaluation and attribution of the patients with depression. In addition, caregivers were asked to complete the Geriatric Depression Scale (GDS) and the Activities of Daily Living (ADL) scale to rate observations of the patients. These questionnaires asked caregivers to choose the answers that best described their patient’s situation.

### Materials

#### Caregivers’ demographic characteristics

The demographic variables assessed in this study were the caregivers’ gender, age, education level, work status, marital status, economic status, health status, relationship with the patient, whether they lived with an individual with depression, average daily care time, and length of time caring for the patient with depression. Health status was assessed through a comprehensive approach, including clinical interviews, information provided by caregivers, review of medical records, and examination of medications. Educational attainment was categorized as elementary school graduate or lower, junior high school graduate, senior high school graduate, or college graduate or higher. Occupation was dichotomized as full-time employment or not. Marital status was dichotomized as married or unmarried. Caregivers’ relationships with the patients were categorized as parent, child, spouse, or other.

#### Hamilton depression rating scale (HAM-D)

We used the HAM-D to measure the severity of depressive symptoms in the patients diagnosed with depression. The HAM-D is administered by researchers who are mainly psychiatrists and other clinical professionals. This study used the Chinese version of HAM-D-17, which is commonly used in Taiwan [3]. The scoring method was divided into three- and five-point scales—8 questions are rated on a 0–2 scale and 9 questions on a 0–4 scale. Total scores were between 0 and 52 points; higher scores indicated more serious depression (≤ 6 is the normal range, 7–17 mild, 18–24 moderate, ≥ 25 severe). Although no research has reported the reliability and validity of the Chinese version of the HAM-D, the scale is based on common physical, cognitive, emotional, dietary, work, and other factors of depression, similar to the DSM description of depression, without cultural differences. In this study, the HAM-D-17 Chinese Version was used as a clinician’s assessment of patients’ depression. The internal consistency of the HAM-D was indicated by a Cronbach’s *α* of 0.91.

#### Geriatric depression scale- proxy assessment (GDS-proxy assessment)

This study was based on a GDS-proxy assessment previously developed [17]. The questionnaire’s design was based on the GDS, which was specially designed to assess depressive symptoms in older patients with depression. The Chinese version of the GDS [18] contains 30 items rated dichotomously (0 = *no such symptom*, 1 = *this symptom*). Higher total scores indicate higher levels of depression. When the Chinese version of the GDS has been used to assess older adults in Taiwan, the internal consistency was *α* = 0.92, the split-half reliability was 0.94, and the recommended cut-off score was 13.

In the caregiver version, the narrative method of questioning was changed relative to caregivers, and the assessment object was changed from “self” to “him/her,” referring to the patient with depression. For example, the proxy version changed the question “Are you often bored?” to read, “Do you think the patient is often bored?” A modified scale was used to assess caregivers’ depression levels.

#### Assessment of care burden

The subjective care burden of caregivers of geriatric patients with depression was assessed using a subjective scale adopted by the Chinese Depression Caregiver Burden Scale (CDCBS) [8], which consists of 14 items distributed across four factors: sadness, anxiety, anger, and guilt. Example items include, “This relationship makes me feel ‘uneasy’ and ‘frustrated’. All questions were assessed on a 5-point Likert scale (0 = *strongly disagree* to 4 = *strongly agree*), with a higher score indicating a higher subjective burden. In our previous study, the subjective care burden subscale of the CDCBS was employed to assess caregivers of patients with depression. The results demonstrated that the subscale possesses satisfactory psychometric properties (Cronbach’s *α* = .83). The present study yielded an internal consistency *α* of .95. Among the 40 participants who enrolled in the follow-up study, the 1-month test-retest reliability was found to be .65. The details of the subjective scale of the CDCBS are displayed in Supplemental Table 1.

#### Assessment of attributional styles

Caregiver attributional styles were evaluated using the Chinese Depression Patient Caregiver Attribution Style Scale (CDPCAS), a novel instrument developed for the present study. This scale was adapted from two related measurements to evaluate caregivers’ attribution of patients’ behaviors. The first questionnaire was the Attribution Scale for Responsibility and Controllability [19], which asks respondents to assess the possibility that the patient is responsible for his/her depression (responsibility attribution) or can control the depression (controllability attribution), which was rated on an 11-point scale (0 = *no*, 10 = *very high*). Higher scores indicated that respondents had stronger beliefs of being responsible for their depression or their ability to control it. An example question was, “How much do you think his suffering from depression is due to his personal responsibility?” The factor analysis results showed that this scale could be divided into two factors—Responsibility Attribution (3 items) and Control Attribution (three items). The internal consistency was Cronbach’s *α* of 0.81 and 0.79 for the two factors, respectively. An additional attribution-style questionnaire was adapted from Polenick and Martire [20] to assess “person-centered attributions.” This questionnaire comprised five items measuring Character Attribution (two items), Controllable Attribution (one item), and Attribution of Intention (two items). Respondents were asked to evaluate the possibility that the patients were responsible for their depression or could control their depression. Items were rated on a 6-point Likert scale (1 = *strongly disagree* to 6 = *strongly agree*). Questions included items related to Character Attribution (2 items), Controllable Attribution (1 item), and Attribution of Intention (2 items). This questionnaire has some shortcomings, including unclear psychometric data, a limited number of items assessing the constructs, and the inclusion of compound concepts in the description.

To develop a comprehensive measure of caregivers’ attributional styles when caring for older adults with depression, items from the aforementioned two questionnaires were combined. Additional items (e.g., “How much do you think his behaviors are consistent with symptoms commonly observed in individuals with depression?”) were also included to capture external attributions, such as attributing patients’ behaviors to their illness or stress. This comprehensive measure, translated into Chinese and scored uniformly on a 6-point Likert scale, aimed to assess caregivers’ attributions across a broad spectrum. This 12-item CDPCAS comprised four subscales: Responsible Attribution, Manipulation Attribution, Controllable Attribution, and Illness and Stress Attribution. Each subscale consisted of three items. Responsible attribution involves caregivers assigning blame for the patient’s condition to the patient themselves. Controllable attribution reflects caregivers’ belief that the patient can exert control over the depressive symptoms. Manipulation attribution involves caregivers perceiving the patient’s symptoms as a means to manipulate or control others. Illness and stress attribution lead caregivers to attribute patient behaviors to underlying symptoms, stress, or physical discomfort. Its construct validity was verified by factor analysis [21]. The internal consistency *α* of the full scale was 0.74. The Cronbach’s α values for Responsible Attribution, Manipulation Attribution, Controllable Attribution, and Illness and Stress Attribution were 0.65, 0.71, 0.76, and 0.60, respectively. The 4-week test-retest reliability of the full-scale questionnaire was 0.73 (*p* < .001), with the test-retest reliability for the four subscales ranging from 0.56 to 0.78 (*p* < .001). In the present study, Cronbach’s *α* for the four subscales were 0.74, 0.78, 0.72, and 0.61, respectively.

### Center of epidemiological studies-depression (CES-D)

We used the CES-D to evaluate caregivers’ level of depression. The CES-D, developed by Radloff et al. [22] was utilized as a comparative measure alongside the HAM-D-17. The CES-D includes 20 items with an internal consistency coefficient of 0.90 [23]. Items were rated on a four-point scale (0 = *rarely occur* to 3 = *frequently occur*), and the total scores totaled 0–60, with 16 as the cut-off score (i.e., 0–15, no depression; 16–20, mild depression; 21–30, moderate depression; > 30, severe depression). The higher the score, the higher the level of depression. Questions included, “I am troubled by things that don’t usually bother me,” “I don’t want to eat; my appetite is very poor,” and “Even with the help of family and friends, I still feel very depressed.” Questions 4, 8, 12, and 16 were reverse-scored. The internal consistency of the CES-D in the patients with depression was 0.89, and the test-retest reliability was 0.78. Internal consistency for caregivers of depression was 0.93.

### Activities of daily living scale, ADL-proxy assessment

Since the condition and level of functional impairment in older adults with depression are important for determining their care needs, we used the Activities of Daily Living Scale to assess the patients’ level of functional impairment. Two types of daily living scales were assessed: (1) the physical or basic activities of daily living scale (ADL) assessing the patients’ ability to use their body, such as sitting, standing, and walking, and (2) functional basic activities (instrumental activities of daily living scale [IADL]) to assess patients’ ability to use tools. The ADL scale contains 10 questions [24]—eating, moving (from bed or wheelchair), personal hygiene, toileting, bathing, walking, going up and down stairs, putting on and taking off clothes, urination control, and defecation control. Each question was evaluated on a 3-point scale (1 = *completely dependent*, 2 = *needing assistance*, and 3 = *completely independent*). Higher scores indicated the patients had better physical activity and daily living abilities. IADL [25] assesses a patient’s ability to use the tools, including shopping, going out, cooking, housework, washing clothes, using the phone, taking medication, and handling finances. Items were rated on a 3-point scale (1 = *completely dependent*, 2 = *needing assistance*, and 3 = *completely independent*). Higher scores indicate better instrumental daily life functioning. The proxy version was used according to the process conducted in “Geriatric Depression Scale- proxy assessment, GDS-proxy assessment.”

### Statistical analysis

IBM SPSS 22.0 (IBM SPSS; Chicago, IL, USA) was used for all analyses. Continuous variables were presented as means and standard deviations. Categorical variables were presented as frequencies and percentages. For the univariate analysis, Pearson’s correlation analysis was used to determine correlations among care burden, age, caregiving time, patients’ symptom severity, caregivers’ ratings of depression, and attributional models. Student’s *t*-test was used to compare the mean differences between the two groups. For the multivariate analysis, hierarchical regression analyses were conducted to determine the predictors of care burden. The variance inflation factor (VIF) was calculated to diagnose collinearity and detect multicollinearity among the independent variables in the regression model. The criterion for severe multicollinearity was VIF > 10, a common rule of thumb, as demonstrated previously [26]. All statistical tests were two-tailed, with *p* < .05 considered statistically significant.

## Results

### Patients and caregivers’ characteristics

The baseline characteristics of the 146 participants are presented in Table 1. Most older adults with depression were women (*n* = 109, 74.7%), with a mean age of 74.3 years (*SD =* 7.1). The average depression severity (HAM-D17) score was 15.7 (*SD =* 5.0), indicating mild to moderate depression. Most caretakers were also women (*n* = 94, 64.4%), with a mean age of 57.7 years (*SD =* 12.9). Caretakers were generally unemployed (*n* = 88, 60.3%) or were engaged in full-time work (*n* = 54, 37.0%), with a significant majority of 112 individuals (76.7%) being married. The mean duration for caregiving was 6.9 years (*SD =* 7.3). The mean scores of CESD, care burden, and attributional model reported by caregivers themselves were 9.3 (*SD =* 9.8), 15.2 (*SD =* 11.9), and 62.9 (*SD =* 10.0), respectively.

**Table 1:** Characteristics of the patients and caregivers (*N* = 146)

	Depression patients	Caregivers
Age, years	74.3 ± 7.1	57.7 ± 12.9
Gender		
Male	37 (25.3%)	52 (35.6%)
Female	109 (74.7%)	94 (64.4%)
Caregiver-patient relationship		
Spouse	-	49 (33.6%)
Child	-	80 (54.8%)
Daughter or son-in-law	-	9 (6.2%)
Other	-	8 (5.5%)
Work status		
None	145 (99.3%)	88 (60.3%)
Part-time	0 (0%)	4 (2.7%)
Full-time	1 (0.7%)	54 (37.0%)
Education level		
Elementary school	55 (37.7%)	17 (11.6%)
Junior high school	36 (24.7%)	17 (11.6%)
High school	30 (20.5%)	41 (28.1%)
College and above	19 (13.0%)	68 (46.6%)
Other/Unknown	6 (4.1%)	3 (2.1%)
Marital status		
Unmarried	1 (0.7%)	28 (19.2%)
Married or live together	108 (74.0%)	112 (76.7%)
Divorced	1 (0.7%)	6 (4.1%)
Widowed	36 (24.6%)	0 (0%)
Main source of income		
Individual	-	46 (31.5%)
Self and other family members	-	74 (50.7%)
Other family members only	-	25 (17.1%)
Others	-	1 (0.7%)
Self-rated economic status		
Affluent	-	2 (1.4%)
Comfortable	-	78 (53.4%)
Moderate	-	61 (41.8%)
Poor	-	5 (3.4%)
Self-perceived health status		
Excellent	-	9 (6.2%)
Good	-	71 (48.6%)
Fair	-	55 (37.7%)
Poor	-	10 (6.8%)
Very poor	-	1 (0.7)
Living together with patients		
Yes	-	88 (60.3%)
No	-	58 (39.7%)
Caregiving duration, years	-	6.9 ± 7.3
***Patient’s symptom severity***		
ADL	92.0 ± 16.5	-
IADL	17.0 ± 6.2	-
Depression level^1^	15.7 ± 5.0	9.3 ± 9.8
***Attributional style***		62.9 ± 10.0
Responsible	-	14.4 ± 4.4
Manipulation	-	11.5 ± 3.8
Controllable	-	12.7 ± 2.5
Illness/stress	-	24.0 ± 6.0
***Care burden***	-	15.2 ± 11.9

Data are presented as N (%) or mean ± SD.

^1^Patient’s depression level was assessed by the HAM-D17 total score; caregivers’ depression level was assessed by the of CES-D total scores.

Care burden, Caregiver’s subjective care burden; CESD, Epidemiological Research Center Depression Scale (caregivers); HAM-D17, Hamilton Depression Rating Scale (patient); ADL/IADL, Activities of Daily Living Scale/ Instrumental Activities of Daily Living Scale (patient).

### Associations between caregivers’ demographics, depression status, attributional style, patients’ symptom severity, and care burden

The results of univariate analysis are shown in Table 2. Caregivers’ care burden was negatively correlated with caregivers’ age (*r* = − .25, *p* = .003), patients’ ADL (*r* = − .17, *p* = .043), and IADL (*r* = − .26, *p* = .002), and positively correlated with caregivers’ depression level (CES-D; *r* = .57, *p* < .001), as well as patients’ depression severity (HAM-D17; *r* = .25, *p* = .003). Caregiver with full time job or higher education had higher caregivers’ burden (mean: 18.3 ± 13.5 vs. 13.1 ± 10.2, *p* = .010 and 17.7 ± 12.0 vs. 13.2 ± 11.5, *p* = .024, respectively). Care burden was also correlated with caregivers’ attributional styles (*r* = .46, *p* < .001), specifically the responsible attribution style (*r* = .17, *p* = .038), manipulation attribution style (*r* = .52, *p* < .001), and illness/stress attribution style (*r* = .40, *p* < .001).

**Table 2:** Association between caregivers’ demographics, depression, attributional style, patients’ severity, and caregiver’s burden

	*N*	Caregivers’ burden		*p*-value
		*r*	mean ± SD	
***Caregivers’ demographics***				
Age, years		-0.25		**0.003**
Gender				0.088
Male	52		12.9 ± 10.8	
Female	94		16.4 ± 12.3	
Caregiver-patient relationship				0.512
Spouse/Child	129		15.4 ± 12.2	
Other	17		13.4 ± 8.7	
Work status				**0.010**
Part time	88		13.1 ± 10.2	
Full time	58		18.3 ± 13.5	
Education level				**0.024**
College and above	68		17.7 ± 12.0	
Other	75		13.2 ± 11.5	
Marital status				0.138
Married or live together	112		14.3 ± 10.9	
Unmarried/Divorced/Widowed	34		18.3 ± 14.2	
Main source of income				0.088
Individual	46		18.0 ± 14.7	
Other	100		13.9 ± 10.1	
Economic status				0.162
Affluent/Comfortable	80		13.9 ± 9.7	
Moderate/Poor	66		16.8 ± 13.9	
Self-perceived health status				0.058
Excellent/Good	80		13.4 ± 9.5	
Fair/Poor/Very Poor	66		17.3 ± 14.0	
Living together with patients				0.411
Yes	88		14.5 ± 12.5	
No	58		16.2 ± 10.9	
Caregiving time (years)	146	0.03		0.735
***Patients’ symptom severity***				
ADL	146	-0.17		**0.043**
IADL	146	-0.26		**0.002**
HAM-D17	146	0.25		**0.003**
***Caregivers’ depression level*** ^***1***^				
CESD	146	0.57		**< 0.001**
***Attributional style***		0.46		**< 0.001**
Responsible	146	0.17		**0.038**
Manipulation	146	0.52		**< 0.001**
Controllable	146	-0.11		0.178
Illness/stress	146	0.40		**< 0.001**

Significant results are shown in bold.

Continuous data are presented as Pearson coefficients, r and categorical data were presented as mean ± SD in each subgroup.

^1^Patient’s depression symptom severity was assessed by the HAM-D17 total score; caregivers’ depression level was assessed by the of CES-D total score.

Care burden, Caregiver’s subjective care burden; CESD, Epidemiological Research Center Depression Scale (caregivers); HAM-D17, Hamilton Depression Rating Scale (patient); ADL/IADL, Activities of Daily Living Scale/ Instrumental Activities of Daily Living Scale (patient).

### Associations between caregivers’ demographics and attributional style

The results of univariate analysis are shown in Supplemental Table 2. The significance of caregivers’ demographics and overall attributional style score was not found, but caregivers’ age had significant negatively correlation with responsible attributional style (*r* = − .25, *p* = .003) and caregiver was female or with full time job had higher manipulation style (mean: 12.0 ± 4.2 vs. 10.7 ± 3.0, *p* = .042 and 12.4 ± 4.3 vs. 10.9 ± 3.4, *p* = .027, respectively).

### Factors associated with care burden

Table 3 presents the results of the hierarchical regression analysis. The predictors of the model showed no significant multicollinearity (all VIFs < 2.74). The results show that *R*^2^ of the models ranged from 0.17 reached 0.57. Each step significantly increased the prediction, especially as CESD added in that step had the greatest improvement in prediction (Model 2 to Model 3: Δ*R*^2^ = 0.16, Δ *F* = 36.10, Δ *p* < .001).

**Table 3:** Hierarchical regression analysis for care burden

	Model 1		Model 2		Model 3		Model 4	
	*β*	*p*-value	*β*	*p*-value	*β*	*p*-value	*β*	*p*-value
**Caregiver’s demographics**								
Gender, male	-0.16	0.068	**-0.23**	**0.008**	**-0.17**	**0.024**	**-0.14**	**0.044**
Age, years	-0.07	0.528	-0.05	0.612	0.05	0.571	0.08	0.365
College and above	0.18	0.058	**0.22**	**0.014**	**0.16**	**0.040**	**0.19**	**0.008**
Work	0.16	0.092	0.15	0.090	**0.18**	**0.029**	0.10	0.163
Married or live together	0.02	0.853	0.11	0.255	0.11	0.209	0.05	0.493
Spouse/Child vs. other	0.07	0.389	0.13	0.097	0.08	0.270	0.07	0.301
Living together	0.07	0.431	0.09	0.321	0.08	0.352	0.10	0.187
Caregiving time	-0.02	0.827	-0.03	0.697	-0.06	0.410	0.00	0.992
Main source of income, Individual	0.05	0.603	0.07	0.406	0.04	0.641	0.06	0.387
Economic status, Poor/Moderate	0.11	0.197	0.12	0.149	0.03	0.652	0.01	0.931
Self-perceived health status, Fair/Poor/Very poor	**0.21**	**0.013**	**0.19**	**0.023**	0.03	0.727	0.00	0.954
***Patients’ symptom severity***								
ADL			0.07	0.574	0.01	0.948	<-0.001	0.998
IADL			**-0.23**	**0.048**	-0.12	0.254	0.02	0.835
HAM-D17			**0.26**	**0.002**	**0.18**	**0.014**	0.07	0.351
***Caregivers’ depression level***								
CESD					**0.49**	**< 0.001**	**0.41**	**< 0.001**
***Attributional style***								
Responsible							-0.01	0.899
Manipulation							**0.29**	**< 0.001**
Controllable							-0.08	0.229
Illness/stress							**0.23**	**0.002**
*R* ^*2*^	0.17		0.29		0.45		0.57	
*Adjusted R* ^*2*^	0.10		0.21		0.38		0.50	
*R Square Change*	0.17		0.12		0.16		0.12	
*F Change*	2.44		7.29		36.10		8.49	
*Change p-value*	**0.009**		**< 0.001**		**< 0.001**		**< 0.001**	

Significant results are shown in bold.

β: Standardized Coefficients of the linear model.

The final model (Model 4) shows that male caregivers had less care burden than female caregivers (*β* = − 0.14, *p* = .044). In addition, caregivers’ education level of college and above (*β* = 0.19, *p* = .008), higher depression on the CES-D (*β* = 0.41, *p* < .001), and higher scores for manipulation attribution style (*β* = 0.29, *p* < .001) and illness/stress attribution style (*β* = 0.23, *p* = .002) were significantly associated with higher care burden. In contrast, patients’ symptom severity was not significantly associated with caregivers’ care burden after controlling for caregivers’ attributional styles (Model 3 and Model 4).

## Discussion

Our study identified several factors associated with higher care burden for caregivers of older patients with depression. Specifically, male caregivers generally reported less care burden than female caregivers, with higher educational levels and caregivers’ own depressive symptoms on the CES-D correlating with an increased care burden. Notably, specific attributional styles, particularly the manipulation and illness/stress attributional styles, were independently associated with increased care burden. Nevertheless, the severity of the patient’s depressive symptoms was not significantly associated with caregivers’ care burden after accounting for attributional style.

A previous study by Yu et al. highlighted the pivotal role of caregivers’ care burden, influencing not only their own mental health but also their caregiving behaviors and attitudes, potentially leading to rejection toward patients [8]. Therefore, attention to caregivers’ care burdens is crucial, necessitating the provision of tailored support and assistance and representing the impetus for this study.

The present study found that when caregivers tended to adopt manipulation and illness/stress attributional styles, their care burden was significantly higher. Similarly, Polenick and Martire [20] surveyed caregivers of older adults with depression and found that more than one-third attributed patients’ depressive symptoms to the patients’ personalities, predicting higher care burdens. In the study by Marguerite et al. [27], which included 79 pairs of caregivers and patients with depression, findings revealed a significant link between the avoidance coping methods of caregivers and anxiety levels for both the caregivers themselves and their patients. Another study found that caregiver burden directly worsens mental health, influenced by personality traits, coping style, and family function. Specifically, neuroticism affects caregiver burden and family functioning, whereas coping style directly influences caregiver burden [28]. The association between caregiver burden and the attributional styles of manipulation and illness/stress may stem from these perspectives exacerbating feelings of helplessness and stress. This amplification can make caregiving tasks appear more daunting and challenging. Offering education on knowledge toward depression and coping mechanisms to caregivers may mitigate this burden by altering their attributional views.

Furthermore, improvements in the caregiver-receiver relationship are proposed to attenuate the care burden effectively [29]. A 20-month follow-up study focusing on 103 fragile older patient-caregiver dyads showed that the mean depression levels and changes in depression of caregivers correlated significantly with attenuated independence [30]. Independence may buffer the influence of care burden on caregivers’ mental and physical symptoms; therefore, enhancing their independence could be effective in attenuating caregivers’ depression levels [31]. A 10-year follow-up study by Lyons et al. [32] with 255 spouses of patients with Parkinson’s disease found that dependency and optimistic characteristics were associated with attenuated caregiver burden.

The interplay between sociodemographic factors, care burden, and the presence of stress in caregivers of individuals with dementia and psychiatric conditions is a focal point of research across different cultural contexts. Our findings indicate that economic status has no significant impact on the burden of caregivers. However, research conducted by De Fazio and others in southern Italy found that caregivers from lower socioeconomic backgrounds, with higher urban living conditions, and of older age, experience greater levels of burden and symptoms of depression [33]. Similarly, Chaparro-Díaz et al. discovered that family caregivers with lower incomes need more social support to alleviate their burden [34]. In addition, we found that caregivers with higher education level had heavier care burden than those who with lower educational level. Oedekoven et al. reported that education level didn’t affect physical strain in informal caregivers, but those with higher education faced more mental burden, potentially due to worries about losing self-fulfillment and autonomy [35]. Nevertheless, another study indicated that the caregiver burden was higher in those with a low education level [36].

Overall, as mentioned above, it is essential to develop interventions that focus on caregivers of older adults with depression. The findings of the present study emphasize the role of caregivers’ attributional styles in care burden, and this may suggest the need for proper strategies, such as increasing educational resources and support networks, to alter the attribution styles of caregivers and ultimately attenuate their care burden. Interventions such as family-focused treatment, as demonstrated in prior research on caregivers of individuals with bipolar disorder [37, 38], have shown promising outcomes. Applying evidence-based study findings could facilitate the development of interventions tailored to caregivers of older adults with depression, allowing for the validation of efficacy.

### Strengths and limitations

Our study provides a detailed examination of how caregivers’ attributional styles influence their perceived care burden when assisting older adults with depression, using robust hierarchical regression analysis and introducing novel assessment scales. However, this study had several limitations. First, the cross-sectional study design does not allow causal inferences, which is the primary limitation in drawing conclusions about cause-effect relationships. Another longitudinal or follow-up study is needed to further validate and investigate the true cause-effect relationship. Second, the study sample consisted of caregivers of older adults diagnosed with depression whose physical condition and mental health details (e.g., onset frequency and duration of depressive symptoms, received antidepressants) were not collected systematically, even though the levels of older patients’ physical deficiency and depression were evaluated using ADL and HAM-D assessments. Excluding the potential influence of different levels of illness on caregivers’ mental and physical wellness is challenging. Third, we used convenience sampling. The sample was recruited from partners who were accompanying patients or those directly introduced by patients.

Additionally, although the actual caregiving reality was identified by questionnaires, a concrete single indicator is lacking to evaluate the caregiving level of the caregiver toward the receiver. Moreover, older patients with depression are sometimes cared for by more than one caregiver (e.g., adult children). Since this study included only one caregiver for one older patient, the current results should be interpreted conservatively, and some caution is necessary when considering whether the current explanations and conclusions are representative of the overall population. Fourth, neither caregivers nor patient comorbidities were collected; therefore, the involvement of other health factors is unknown. Finally, some older adults with depression in Taiwan are cared for by employed caregivers. However, these studies were excluded from the study population; therefore, caution is needed regarding the interpretation and inferences of this study.

## Conclusions

This study highlights the influence of caregivers’ attributional styles on patients’ behavior and symptoms, particularly the manipulation and illness/stress attributional styles, which significantly increase caregivers’ care burden. These findings identified the need for educational resources to change the attribution style, accompanied by support systems and accessible mental health services for caregivers, to potentially reduce the care burden.

### Electronic supplementary material

Below is the link to the electronic supplementary material.

Supplementary Material 1

## Data Availability

No datasets were generated or analysed during the current study.
