# Supplementary material for: Attributional styles are associated with care burden in geriatric depression: older adults and their caregivers in Taiwan
Source: Aging Clin Exp Res. 2024 May 8;36(1):106. doi: 10.1007/s40520-024-02762-2 (PMC11076353; doi:10.1007/s40520-024-02762-2)
Supplement: Supplementary file 1 — Supplementary Material 1 [file 40520_2024_2762_MOESM1_ESM.docx]

**Supplemental Table 1.** The Subjective scale of The Chinese Depression Caregiver Burden Scale (CDCBS).

| Please answer the following questions based on your experiences with the person you are caring for in the past month (including today).  Please read each statement carefully and check the box that best describes your situation. | | | | | |
| --- | --- | --- | --- | --- | --- |
|  | Never | Rarely | Sometimes | Often | Almost always |
|  | 0% | 25% | 50% | 75% | 100% |
| 1. **Makes me feel “uneasy”** |  |  |  |  |  |
| 1. **Makes me feel “frustrated”** |  |  |  |  |  |
| 1. **Makes me feel “furious”** |  |  |  |  |  |
| 1. **Makes me feel “afraid”** |  |  |  |  |  |
| 1. **Makes me feel “nervous”** |  |  |  |  |  |
| 1. **Makes me feel “remorseful”** |  |  |  |  |  |
| 1. **Makes me feel “sad”** |  |  |  |  |  |
| 1. **Makes me feel “impatient”** |  |  |  |  |  |
| 1. **Makes me feel “**upset**”** |  |  |  |  |  |
| 1. **Makes me feel “guilty”** |  |  |  |  |  |
| 1. **Makes me feel “annoyed”** |  |  |  |  |  |
| 1. **Makes me feel “ashamed”** |  |  |  |  |  |
| 1. **Makes me feel “angry”** |  |  |  |  |  |
| 1. **Makes me feel “dissatisfied”** |  |  |  |  |  |

| Supplemental table 2. The association between caregivers' demographics and attributional style. | | | | | | | | | | | | | | | | | | | |
| --- | --- | --- | --- | --- | --- | --- | --- | --- | --- | --- | --- | --- | --- | --- | --- | --- | --- | --- | --- |
|  | Attributional Style | | p-value |  | Responsible | | p-value |  | Manipulation | | p-value |  | Controllable | | p-value |  | Illness/stress | | p-value |
|  | *r* | mean ± SD |  |  | *r* | mean ± SD |  |  | *r* | mean ± SD |  |  | *r* | mean ± SD |  |  | *r* | mean ± SD |  |
| ***Caregivers' Demographics*** |  |  |  |  |  |  |  |  |  |  |  |  |  |  |  |  |  |  |  |
| Age | 0.05 |  | 0.564 |  | -0.25 |  | **0.003** |  | -0.16 |  | 0.058 |  | 0.15 |  | 0.069 |  | -0.07 |  | 0.416 |
| Sex |  |  | 0.701 |  |  |  | 0.297 |  |  |  | **0.042** |  |  |  | 0.936 |  |  |  | 0.266 |
| Male |  | 63.3 ± 9.0 |  |  |  | 14.9 ± 4.0 |  |  |  | 10.7 ± 3.0 |  |  |  | 12.8 ± 2.3 |  |  |  | 24.9 ± 5.2 |  |
| Female |  | 62.6 ± 10.6 |  |  |  | 14.1 ± 4.6 |  |  |  | 12.0 ± 4.2 |  |  |  | 12.7 ± 2.7 |  |  |  | 23.9 ± 5.7 |  |
| Caregiver-patient relationship |  |  | 0.457 |  |  |  | 0.483 |  |  |  | 0.586 |  |  |  | 0.708 |  |  |  | 0.557 |
| Spouse/Child |  | 62.7 ± 10.2 |  |  |  | 14.3 ± 4.4 |  |  |  | 11.5 ± 3.8 |  |  |  | 12.8 ± 2.6 |  |  |  | 24.2 ± 5.6 |  |
| Other |  | 64.6 ± 8.9 |  |  |  | 15.1 ± 3.9 |  |  |  | 12.0 ± 4.4 |  |  |  | 12.5 ± 2.1 |  |  |  | 25.0 ± 5.1 |  |
| Work status |  |  | 0.400 |  |  |  | 0.426 |  |  |  | **0.027** |  |  |  | 0.910 |  |  |  | 0.621 |
| Part time |  | 62.3 ± 9.8 |  |  |  | 14.6 ± 4.2 |  |  |  | 10.9 ± 3.4 |  |  |  | 12.7 ± 2.5 |  |  |  | 24.1 ± 5.5 |  |
| Full time |  | 63.7 ± 10.4 |  |  |  | 14.0 ± 4.6 |  |  |  | 12.4 ± 4.3 |  |  |  | 12.8 ± 2.6 |  |  |  | 24.5 ± 5.7 |  |
| Education level |  |  | 0.380 |  |  |  | 0.088 |  |  |  | 0.498 |  |  |  | 0.625 |  |  |  | 0.656 |
| College and above |  | 62.0 ± 10.3 |  |  |  | 13.6 ± 4.7 |  |  |  | 11.3 ± 3.3 |  |  |  | 12.6 ± 2.6 |  |  |  | 24.4 ± 6.0 |  |
| Other |  | 63.5 ± 9.9 |  |  |  | 14.9 ± 4.1 |  |  |  | 11.8 ± 4.3 |  |  |  | 12.8 ± 2.5 |  |  |  | 24.0 ± 5.0 |  |
| Marital status |  |  | 0.874 |  |  |  | 0.752 |  |  |  | 0.408 |  |  |  | 0.469 |  |  |  | 0.768 |
| Married or live together |  | 62.8 ± 10.0 |  |  |  | 14.4 ± 4.3 |  |  |  | 11.4 ± 3.7 |  |  |  | 12.8 ± 2.5 |  |  |  | 24.2 ± 5.8 |  |
| Unmarried/Divorce/Widowed |  | 63.1 ± 10.3 |  |  |  | 14.1 ± 4.7 |  |  |  | 12.0 ± 4.3 |  |  |  | 12.5 ± 2.5 |  |  |  | 24.5 ± 4.9 |  |
| Main source of income |  |  | 0.613 |  |  |  | 0.588 |  |  |  | 0.929 |  |  |  | 0.303 |  |  |  | 0.348 |
| Only selves |  | 63.5 ± 10.2 |  |  |  | 14.1 ± 4.5 |  |  |  | 11.5 ± 4.2 |  |  |  | 13.1 ± 2.8 |  |  |  | 24.9 ± 4.8 |  |
| Other |  | 62.6 ± 10.0 |  |  |  | 14.5 ± 4.3 |  |  |  | 11.5 ± 3.7 |  |  |  | 12.6 ± 2.4 |  |  |  | 24.0 ± 5.9 |  |
| Economic status |  |  | 0.221 |  |  |  | 0.610 |  |  |  | 0.145 |  |  |  | 0.671 |  |  |  | 0.565 |
| Rich/Well-off |  | 62.0 ± 9.2 |  |  |  | 14.2 ± 4.6 |  |  |  | 11.1 ± 3.3 |  |  |  | 12.7 ± 2.1 |  |  |  | 24.0 ± 5.4 |  |
| General/Poor |  | 64.0 ± 11.0 |  |  |  | 14.6 ± 4.2 |  |  |  | 12.0 ± 4.4 |  |  |  | 12.8 ± 3.0 |  |  |  | 24.5 ± 5.7 |  |
| Self-perceived health status |  |  | 0.418 |  |  |  | 0.748 |  |  |  | 0.228 |  |  |  | 0.910 |  |  |  | 0.759 |
| Very good/Good |  | 62.3 ± 10.0 |  |  |  | 14.3 ± 4.5 |  |  |  | 11.2 ± 3.4 |  |  |  | 12.7 ± 2.4 |  |  |  | 24.1 ± 6.0 |  |
| Fair/Bad/Vary bad |  | 63.6 ± 10.2 |  |  |  | 14.5 ± 4.2 |  |  |  | 12.0 ± 4.3 |  |  |  | 12.8 ± 2.7 |  |  |  | 24.4 ± 5.0 |  |
| Whether living together |  |  | 0.989 |  |  |  | 0.065 |  |  |  | 0.489 |  |  |  | 0.120 |  |  |  | 0.098 |
| Live together |  | 62.9 ± 10.1 |  |  |  | 14.9 ± 4.1 |  |  |  | 11.3 ± 3.9 |  |  |  | 13.0 ± 2.3 |  |  |  | 23.6 ± 5.8 |  |
| Do not live together |  | 62.9 ± 10.1 |  |  |  | 13.5 ± 4.7 |  |  |  | 11.8 ± 3.7 |  |  |  | 12.3 ± 2.8 |  |  |  | 25.2 ± 5.1 |  |
| Caregiving time (years) | -0.06 |  | 0.458 |  | -0.02 |  | 0.776 |  | 0.04 |  | 0.668 |  | 0.05 |  | 0.573 |  | -0.14 |  | 0.093 |
| Significant results were shown in bold. Continuous data are presented as Pearson coefficients, r and categorical data were presented as mean ± SD in each subgroup. | | | | | | | | | | | | | | | | | | | |
